# ELS live surgery: a developing story

**DOI:** 10.1007/s00405-018-05278-4

**Published:** 2019-01-17

**Authors:** Frederik G. Dikkers, Manuel Bernal-Sprekelsen, J. Peter Klussmann, Christian Sittel, Witold Szyfter

**Affiliations:** 1 Department of Otorhinolaryngology, Amsterdam UMC, University of Amsterdam, Meibergdreef 9, Amsterdam, The Netherlands; 2 Department of Otorhinolaryngology, University of Valencia, Valencia, Spain; 3 Department of Otorhinolaryngology, Head and Neck Surgery, Medical Faculty, University of Cologne, Cologne, Germany; 4 Department of Otorhinolaryngology, Klinikum Stuttgart, Stuttgart, Germany; 5 Department of Otolaryngology and Laryngological Oncology, University of Medical Sciences, Poznan, Poland

**Keywords:** Live surgery, Phonosurgery, Laryngeal malignancy, Recurrent respiratory papillomatosis

## Abstract

**Introduction:**

Since 2015, the European Laryngological Society (ELS) has organized on a yearly basis the European Laryngological Live Surgery Broadcast. The goal of this paper is to demonstrate the increasing worldwide audience.

**Material and methods:**

The number of individual computers logged in, number of estimated audience, and number of countries with an active audience were calculated and compared to the numbers in 2015.

**Results:**

In 2018, 19 live interventions were performed in three parallel sessions. The surgeons worked in 10 departments in 8 different countries. The number of individual computers logged in increased from 1000 in 2015 to 16000 in 2018. The estimated audience increased from 3000 to 32000 visitors. The number of countries with an active audience increased from 52 to 91.

**Discussion:**

The amount of computers logged in is increasing year by year. The audience was presenting despite inconvenient broadcasting times, highlighting the educational importance. The teaching aspect remains visible on videos of this year’s and previous year’s interventions. They can be seen on website http://els.livesurgery.net/home.php. The organization of the European Laryngological Live Surgery Broadcast concurs to the idea that live broadcast of laryngologic surgery is feasible and attractive. Therefore, the ELS is going to continue to organize additional European Laryngological Live Surgery Broadcasts in the future.

In 2015, the first European Laryngological Live Surgery Broadcast took place. By then, participating surgeons came from eight hospitals in seven countries. Following that day, the organization has wondered who was actually benefiting from the broadcast [1]. Positive feedback of the first broadcast inspired the European Laryngological Society (ELS) to continue on an annual basis. In November 2018, it was time for the 4th European Laryngological Live Surgery Broadcast.

As usual, viewing of the broadcast was free of charge. In the organization of the broadcast, the potential audience was addressed by announcing the event to all members of the ELS, and by emailing all over the world making use of address lists of the sponsors. In addition, participants and ELS members were encouraged to contact address lists of their countries’ scientific societies.

In 2018, 19 live interventions were performed in three parallel sessions. The surgeons worked in ten departments in eight different countries (Table 1). Like in the previous 3 years, the interventions were moderated from Poznań Supercomputing and Networking Center (PSNC) in Poznań, Poland, where five moderators were actively following all surgeries. The extensive technical requirements of the broadcast have been addressed before [1].

**Table 1:** List of surgeons, displayed alphabetically by the name of their country

Prof. A. Giovanni (Marseille, France)
Prof. S. Lang (Essen, Germany)
Prof. C. Wittekindt (Giessen, Germany)
Prof. R. Puxeddu (Cagliari, Italy)
Prof. M. Remacle (Luxembourg, Luxembourg)
Dr. T. Langeveld (Leiden, The Netherlands)
Prof. M. Wierzbicka (Poznań, Poland)
Prof. M. Quer (Barcelona, Spain)
Prof. I. Vilaseca (Barcelona, Spain)
Mr. R. Simo (London, UK)

Again, moderators had audible live connections with the surgeons, enhancing visibility of relevant parts of the respective interventions. Moderators were further commenting on important and critical steps of the surgery and putting forth questions to the surgeons to help in clarifying procedures. At the final end of the session, which took place from 09:00 to 16:00 CET (GMT + 1), a discussion was conducted. All surgeons were present in their respective surgical theaters, ready to answer questions of the audience. The audience was encouraged to send questions to an email address that was repeatedly displayed.

The audience increased year by year. In the first year, we had some 1000 individual computers logged in from 52 countries, with an estimated audience of 3000 visitors. By the time of our recent fourth session, we had some 16,000 individual computers logged in from 91 countries in all continents with an estimated audience of 32,000 visitors. The list of countries with an active audience is displayed on http://els.livesurgery.net/. During the past years, we have seen an annual increase of visitors from an annual increase of visiting countries. In addition, there is an increasing average number of visitors per country (Fig. 1).

**Fig. 1:**
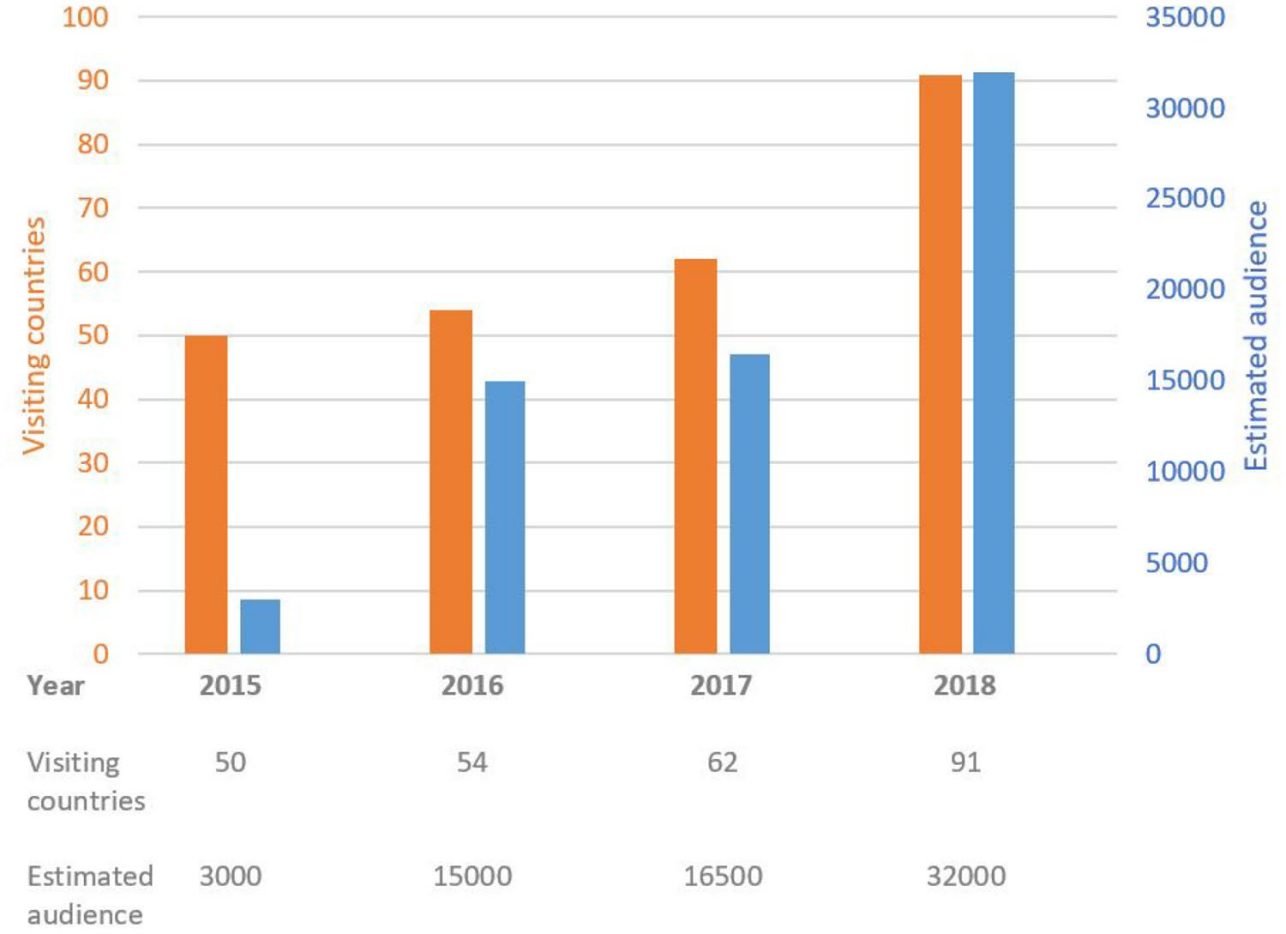
Number of visiting countries and number of estimated audience logged in to ELS Live Surgery Broadcast per year

During broadcast, the audience was encouraged to supply questions to the moderators and surgeons. Questions were received from all over the world—initially from the Far East and China, later more from European countries. The subjects of the questions were especially dealing with two problems: laryngeal malignancies, and recurrent respiratory papillomatosis. That is in contrast with earlier experiences, where phonosurgery was the topic of the majority of questions. The most relevant questions were summarized and discussed.

Like in earlier years, we have been considering the patient side of the broadcast. However, no new perspectives were presented to our earlier point of view [1]. For the surgeons, the fact that thousands of visitors at the same time were watching their performance, of course, induces stress. However, it is impossible to measure or calculate the amount. In the 2018 session, we had unexpected bleeding, slipping of a laryngeal suture, etc. For the audience, it was of utmost importance to learn how to deal with it. The teaching aspect remains visible on videos of this year’s and previous year’s interventions. They can be seen on website http://els.livesurgery.net/home.php.

The amount of computers logged in is increasing year by year. It is interesting to see that in the Western European morning we had visitors from China, Australia and New Zealand, but also from the USA, especially from the Boston area. Later during the broadcast, most other countries from the America’s had visitors as well, as is shown on Fig. 2. This can easily be explained by obvious difference in time zones. This implies that the session might be broadcasted at inconvenient times. In China, the time of broadcast is convenient (late afternoon till midnight), while in the Atlantic USA the broadcast starts at 03:00 AM. However, we encourage departments to reserve dedicated time for staff viewing. In so doing, laryngological procedures can be discussed in the group, which allows for efficient surgical education and standardization within the department at no charge and without travelling.

**Fig. 2:**
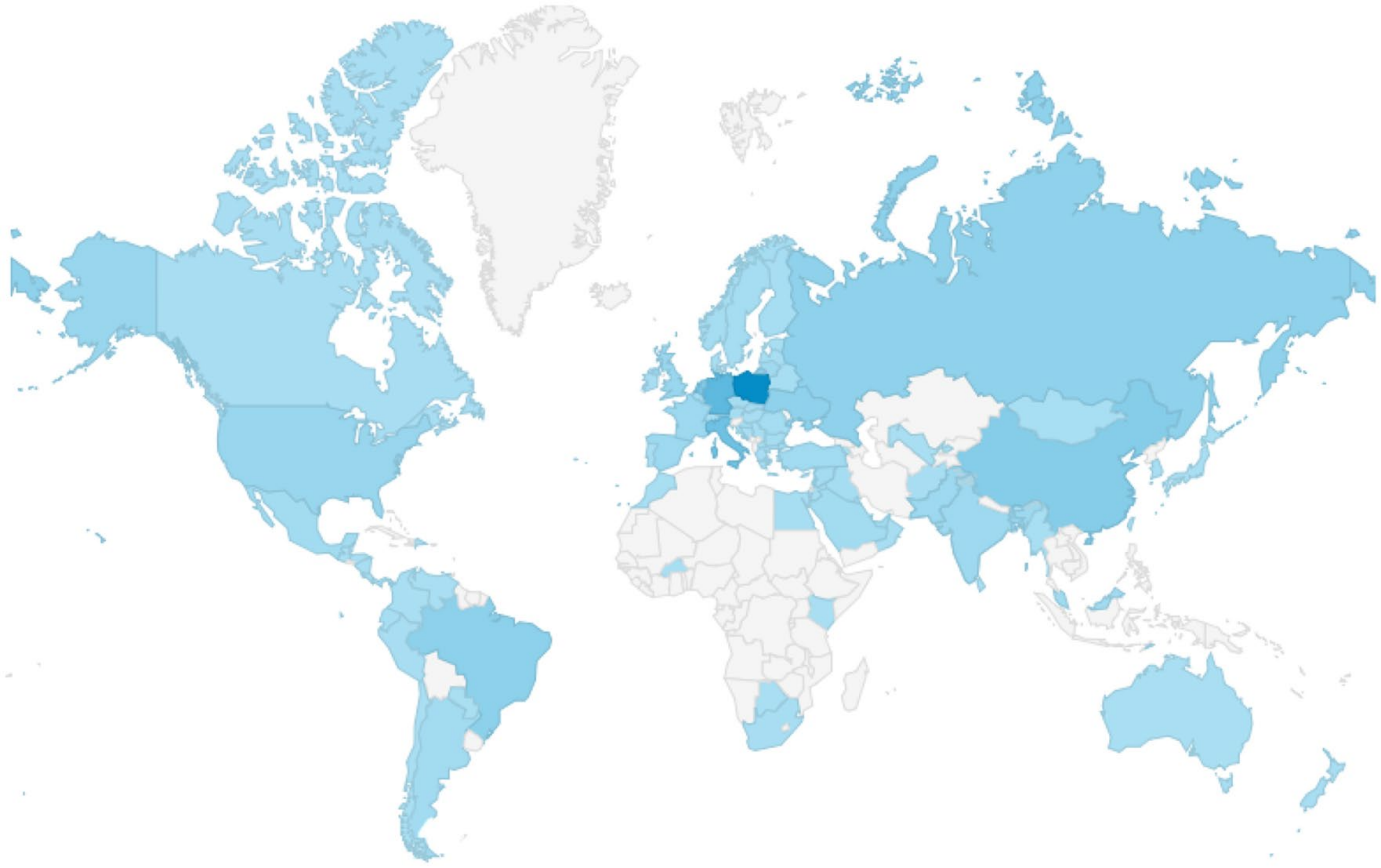
Countries with audience during the 4th European Laryngological Live Surgery Broadcast. From light blue to dark blue: different colors according to the amount of viewers (dark blue is most viewers). White: no viewers

Altogether, the organization of the European Laryngological Live Surgery Broadcast concurs to the idea that live broadcast of laryngologic surgery is feasible and attractive too, where the benefits for education outweigh the potential drawbacks for the consented patient. Therefore, the ELS is going to continue to organize additional European Laryngological Live Surgery Broadcasts in the future.

## References

[1] Dikkers FG, Klussmann JP, Bernal-Sprekelsen M, Mazurek C, Szyfter W (2016). Live surgery broadcast: who is benefiting?. Eur Arch Otorhinolaryngol.

